# Automated sleep staging on reduced channels in children with epilepsy

**DOI:** 10.3389/fneur.2024.1390465

**Published:** 2024-05-10

**Authors:** Renee Proost, Elisabeth Heremans, Lieven Lagae, Wim Van Paesschen, Maarten De Vos, Katrien Jansen

**Affiliations:** ^1^Pediatric Neurology Department, University Hospitals Leuven, KU Leuven, Leuven, Belgium; ^2^Department of Electrical Engineering (ESAT), STADIUS Center for Dynamical Systems, Signal Processing and Data Analytics, KU Leuven, Leuven, Belgium; ^3^Neurology Department, University Hospitals Leuven, KU Leuven, Leuven, Belgium

**Keywords:** children, epilepsy, REM sleep, automated sleep staging, machine learning

## Abstract

**Objectives:**

This study aimed to validate a sleep staging algorithm using in-hospital video-electroencephalogram (EEG) in children without epilepsy, with well-controlled epilepsy (WCE), and with drug-resistant epilepsy (DRE).

**Methods:**

Overnight video-EEG, along with electrooculogram (EOG) and chin electromyogram (EMG), was recorded in children between 4 and 18 years of age. Classical sleep staging was performed manually as a ground truth. An end-to-end hierarchical recurrent neural network for sequence-to-sequence automatic sleep staging (SeqSleepNet) was used to perform automated sleep staging using three channels: C4-A1, EOG, and chin EMG.

**Results:**

In 176 children sleep stages were manually scored: 47 children without epilepsy, 74 with WCE, and 55 with DRE. The 5-class sleep staging accuracy of the automatic sleep staging algorithm was 84.7% for the children without epilepsy, 83.5% for those with WCE, and 80.8% for those with DRE (Kappa of 0.79, 0.77, and 0.73 respectively). Performance per sleep stage was assessed with an F1 score of 0.91 for wake, 0.50 for N1, 0.83 for N2, 0.84 for N3, and 0.86 for rapid eye movement (REM) sleep.

**Conclusion:**

We concluded that the tested algorithm has a high accuracy in children without epilepsy and with WCE. Performance in children with DRE was acceptable, but significantly lower, which could be explained by a tendency of more time spent in N1, and by abundant interictal epileptiform discharges and intellectual disability leading to less recognizable sleep stages. REM sleep time, however, significantly affected in children with DRE, can be detected reliably by the algorithm.

**Clinical trial registration**: ClinicalTrials.gov, identifier NCT04584385.

## 1 Introduction

A bidirectional relationship between sleep and epilepsy has long been considered and it has slowly gained more attention in recent years. Most of the literature on this topic comes from studies in adults (1), although an increasing amount of pediatric literature is emerging (2–4). There is a need to better understand the complex interaction between all sleep and epilepsy-related factors. If we want to study this on a larger scale, we need automated sleep staging. The golden standard of manual sleep staging according to the American Academy of Sleep Medicine (AASM) guidelines is labor-intensive and time-consuming (5). Moreover, the question arises as to whether automated sleep staging algorithms can become more reliable than human scoring over time. Inter-rater reliability of human sleep stage scoring has been studied in the adult population without epilepsy, but not in children (6–8). As classical sleep architecture is disturbed in children with drug-resistant epilepsy (DRE) and intellectual disability (2, 4), we assume that inter-rater reliability will be lower in children without epilepsy or with well-controlled epilepsy (WCE). Although sleep parameters such as the qualitative presence of spindles and K-complexes are reported on video-EEG, sleep staging is not part of standard investigations in current childhood epilepsy practice. However, from research, we know that even in children with WCE, sleep architecture does change (2), and decreased rapid eye movement (REM) sleep time is a consistent finding in children with DRE (4). These changes could be therapeutic targets, to impact the vicious cycle between sleep and epilepsy and improve, for example, daytime sleepiness, a well-known complaint in children with DRE.

An automated sleep staging algorithm could serve various purposes in clinical practice. First, children coming for overnight video EEGs can be screened for sleep disturbances. For instance, children with epilepsy often have comorbid parasomnias, which could be more precisely classified after sleep staging (2). Second, it could be beneficial to use automated sleep staging in the presurgical work-up in children with epilepsy. Since interictal epileptiform discharges are known to focalize during REM sleep, the irritative zone could be delineated more easily (9). Finally, it can facilitate long-term sleep monitoring, which can help overcome the first-night effect and reveal ultradian rhythms (10). In such cases, at-home monitoring with a wearable device featuring limited channels would be ideal. In addition to clinical use, automated sleep staging algorithms have the potential to accelerate sleep research (11). It will also help to process larger sample sizes and improve the reliability of staging.

Most sleep staging algorithms were developed on adult datasets, although the performance of state-of-the-art algorithms on pediatric populations provided good test results as well (12, 13). The pediatric databases used by previous studies included either healthy children or children with obstructive sleep apnea syndrome (OSAS), but no other sleep disorders or neurologic disorders were included, to our knowledge. Recent sleep staging methods predominantly rely on deep learning, particularly deep neural networks employing a sequence-to-sequence approach, which is indispensable for accurate sleep staging. These models are capable of taking into account the long-range context of the epoch that is being scored, comparable to how a human would visually score sleep (11). The most widely used network architectures are recurrent neural networks and convolutional neural networks (14).

In this study, we investigated the performance of an end-to-end hierarchical recurrent neural network for sequence-to-sequence automatic sleep staging (SeqSleepNet) using video-EEG data, with a limited number of electrodes, in a cohort of children without epilepsy, children with WCE, and children with DRE. We performed automated sleep staging on a limited number of electrodes, instead of using full scalp EEG, to investigate its usability for at-home monitoring.

## 2 Methods

### 2.1 Recruitment

Between March 2021 and January 2023, children aged 4 to 18 years who were admitted to the pediatric epilepsy monitoring unit (EMU) for 24-h video-EEG were invited to participate. This included children with DRE, WCE, or children without epilepsy who were admitted to the EMU to either exclude epilepsy or for other reasons. DRE was defined as “failure of adequate trials of two tolerated and appropriately chosen and used anti-seizure medication (ASM) schedules (whether as monotherapies or in combination) to achieve sustained seizure freedom,” according to the International League Against Epilepsy (ILAE) (15). Informed consent was given by all parents before the measurements and an assent was completed in children older than 11 years, if intellectually able. The study was approved by the ethics commission (S64658) of the Catholic University of Leuven, Belgium. This study was part of a larger project called “Advanced EEG Technology in Childhood Epilepsy” (ClinicalTrials.gov NCT04584385). Full scalp video-EEG used a Schwarzer EEG amplifier (O.S.G. Belgium) and Ag/AgCl cup electrodes (Ambu Neuroline Cup, Ambu, Denmark). Twenty-one electrodes were placed using the 10–20 system. Furthermore, extra electrodes for eye movements, electrooculogram (EOG), and chin electromyogram (EMG) were added to make sleep staging possible. Impedance was ≤5 kΩ at the beginning of the measurement. In addition, multiple patient and epilepsy-related characteristics were obtained from the EEG data and the patients’ records.

In total, 182 children were recruited for the study. In six children, EOG and chin EMG data were missing, and hence, sleep stages were manually scored in 176 children: 47 children without epilepsy, 74 with WCE, and 55 with DRE.

### 2.2 Manual sleep stage annotation

We used BrainRT software (O.S.G. Belgium) to visualize video-EEG data. Manual scoring was performed by RP, a certified somnologist-technologist by the European Sleep Research Society, according to the most recent AASM guidelines (5). In children with epilepsy, some additional decisions were made for consistent scoring: When no activity without interictal epileptiform discharges (IEDs) was present, the frequency of the discharges was used to classify the sleep stage: e.g. < 2.5 Hz and > 70 mV was scored as N3. Furthermore, in children with epileptic encephalopathies, the presence of low chin tone together with rapid eye movements (REM) was classified as REM sleep, even in the presence of slow waves instead of low amplitude mixed frequency (LAMF). Similar to children with epileptic encephalopathies, those with LAMF do not exhibit normal background rhythms during wakefulness, nor do they present with LAMF during REM sleep. It was particularly noted while scoring if there were subjectively abundant IEDs and when sleep scoring was particularly difficult.

### 2.3 Algorithm

Automated sleep stage scoring was performed with a state-of-the-art deep learning algorithm for sleep staging called SeqSleepNet (16). After segmenting the physiological signals into segments of 30 s, we transformed each segment into a time-frequency representation. SeqSleepNet takes these time-frequency representations as input and returns probabilities for each of the five sleep stages. This neural network architecture uses a sequence-to-sequence prediction framework, which means it learns the dependencies between consecutive segments. Hence, it is fed with a sequence of multiple consecutive segments and it returns the corresponding sequence of predictions. We used sequences of size *N* = 10 in this study.

We pre-trained SeqSleepNet on the Montreal Archive of Sleep Studies (MASS), with a dataset of 200 healthy adults (17), using three channels: the C4-A1 channel, the EOG (ROC-LOC), and the chin EMG. Sleep staging rules (5) are the same for adults and children, so we expect an algorithm trained on adults to achieve a fair performance in a pediatric population. This approach was further motivated by the scarcity of publicly available pediatric polysomnography datasets. However, small age-related changes in the EEG may result in a suboptimal performance when training on an adult dataset. Therefore, we fine-tuned the algorithm to the pediatric population, hypothesizing that this would correct for age-related differences.

We first evaluated the pre-trained version of the algorithm on the pediatric patients. The pre-trained algorithm was then fine-tuned to the pediatric dataset using the same three channels and the sleep stage labels of the manual scoring. This was achieved with a supervised transfer learning approach. In order to evaluate the performance of the fine-tuned algorithm, we performed 15-fold cross-validation by randomly dividing the dataset into 15 groups of 11–12 patients each. At every iteration of the cross-validation, all except one group were used for training (fine-tuning), and the remaining group was used for testing.

### 2.4 Data analysis

Agreement between the algorithm and manual hypnogram was evaluated for the 5-stage hypnogram (wake/N1/N2/N3/REM). Accuracy was defined as the percentage of epochs correctly scored by the algorithm compared to the manually scored hypnogram. Kappa was calculated as the agreement between the two hypnograms corrected for agreement by chance (18). The sensitivity [true positives/(true positives + false negatives)] and specificity [true negatives/(true negative + false positive)] were calculated per sleep stage. The F1 score was calculated as the harmonic mean of sensitivity and specificity [2x(specificityxsensitivtiy)/(specificity+sensitivity)].

Between-group differences for continuous data were tested using either a two-tailed t-test (with unequal variance) for normally distributed data or the Mann–Whitney U for non-normally distributed data. The chi-square test was performed for categorical data. Simple and multiple linear regression was performed to predict continuous outcomes. *p*-values less than 0.05 were considered significant.

## 3 Results

### 3.1 Overall accuracy

The accuracy of the pre-trained algorithm was 78.1% in children without epilepsy, 74.9% in children with WCE, and dropped to 63.8% in children with DRE. After re-training, the accuracy increased to 84.7% (Kappa 0.79), 83.5% (Kappa 0.77), and 80.8% (Kappa 0.73) respectively. All performance results are summarized in Table 1.

**Table 1:** Performance of SeqSleepNet trained on adult dataset, and fine-tuned on the children’s dataset.

	Pre-trained		Fine-tuned	
	Accuracy (%)	Kappa score	Accuracy (%)	Kappa score
No epilepsy	78.1	0.697	84.7	0.789
Well-controlled epilepsy	74.9	0.653	83.5	0.769
Drug-resistant epilepsy	63.8	0.531	80.8	0.726

Accuracy and Kappa scores are reported and segregated by group.

### 3.2 Performance for different sleep stages

**Figure 1:**
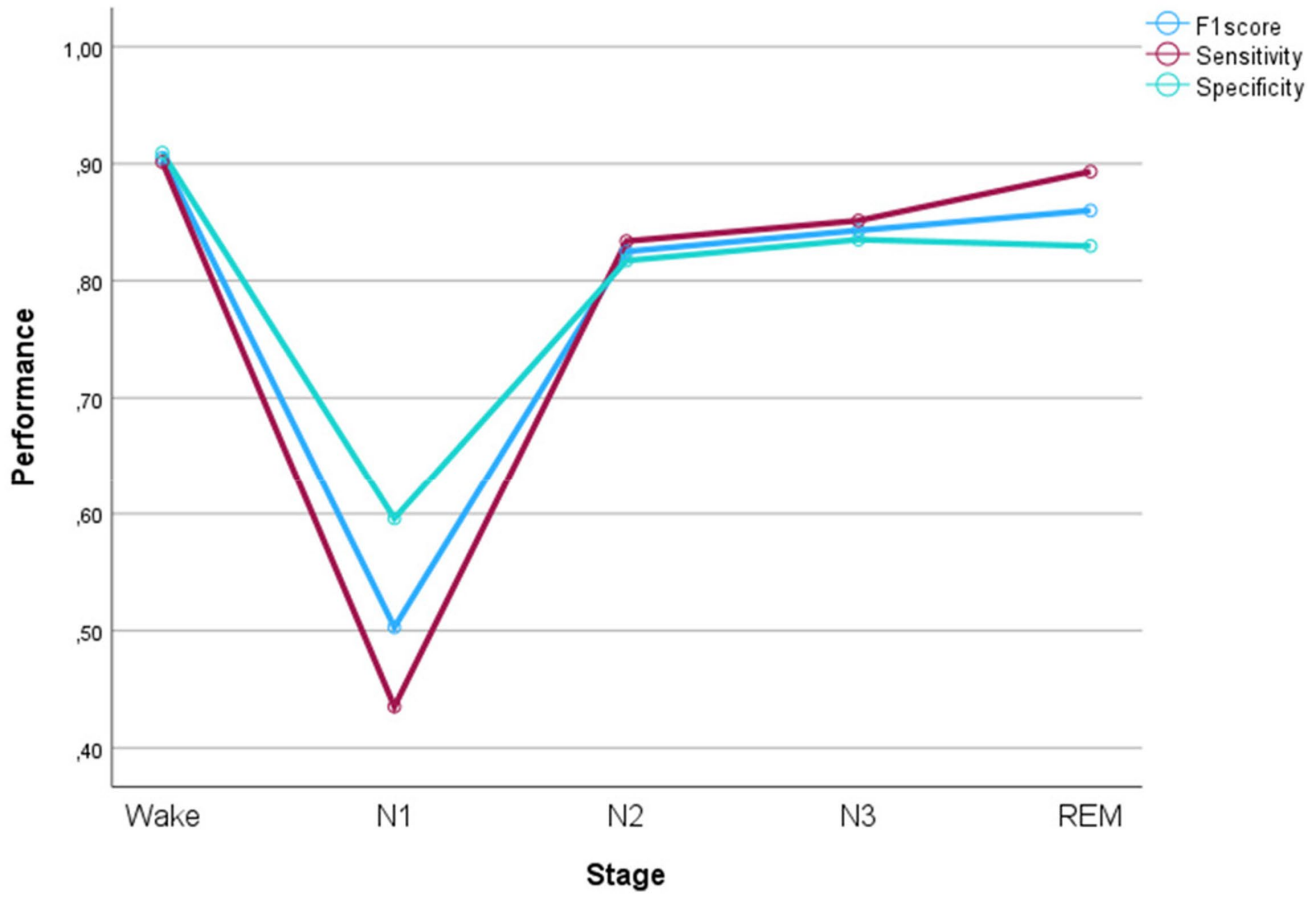
Performance metrics per sleep stage. Sensitivity: true positives/(true positives + false negatives), specificity: true negatives/(true negative + false positive), F1 score: 2x(specificityxsensitivtiy)/(specificity+sensitivity).

The performance of the fine-tuned algorithm was evaluated separately for the wake and all sleep stages. The sensitivity was best in wake and REM sleep, while the precision was best for wake and N3. Both metrics were the lowest for N1 by a large margin. The harmonic mean of the sensitivity and precision, the F1-score, was 0.905 for wake, 0.503 for N1, 0.825 for N2, 0.843 for N3, and 0.860 for REM. The performance results per sleep stage are illustrated in Figure 1. The confusion matrix is shown as a heat map in Supplementary Figure S1.

### 3.3 Interpretation of between-group differences

**Figure 2:**
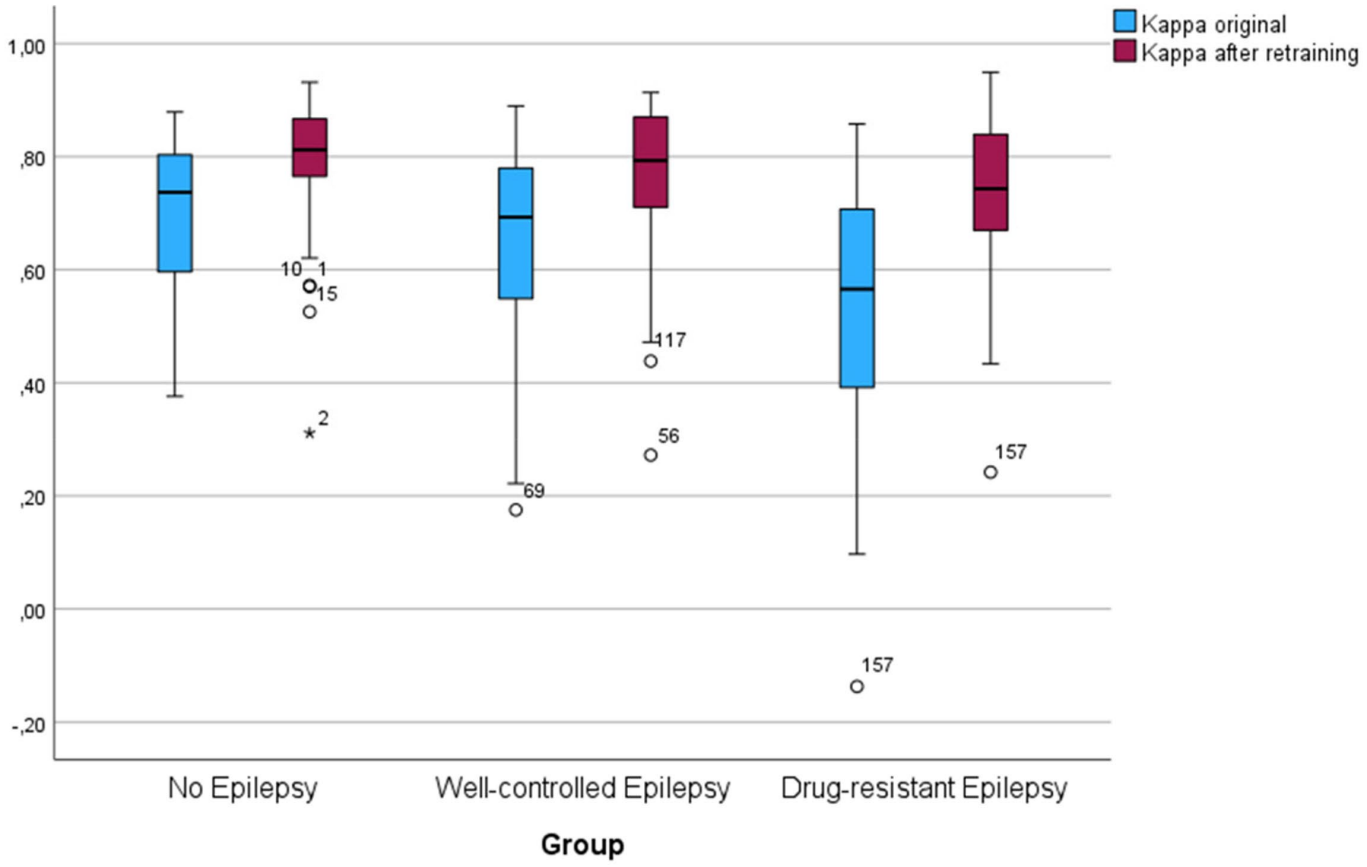
Box-plot of Kappa scores per group before and after fine-tuning the algorithm on the children’s dataset.

**Figure 3:**
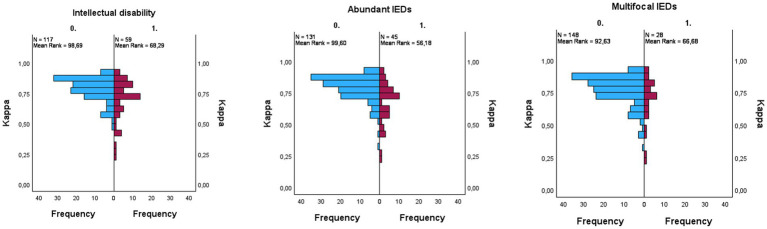
Univariate analyses conducted using the Mann–Whitney U test. Kappa per subgroup: intellectual disability ((*p* < 0.001), abundant interictal epileptiform discharges (IEDs) ((*p* < 0.001), multifocal IEDs ((*p* = 0.013). Blue = not present, purple = present.

Patients with drug-resistant epilepsy Had significantly lower kappa scores compared To patients without epilepsy and well-controlled epilepsy (PDRE-control = 0.005, PDRE-WCE = 0.039). The kappa scores, before and after re-training, Per group Are illustrated In Figure 2. In order To identify The reasons for lower scoring performance In The DRE group, We looked at some sleep and patient characteristics. Initially, N1 staging Was difficult for The algorithm; a difference between groups for time spent In N1 could explain The different performances. Although there Was indeed a trend towards more time spent In N1 for The DRE group, this Was Not statistically significant (PDRE-WCE = 0.066). In The drug-resistant group, there were significantly more patients with intellectual disability and multifocal and/or abundant IEDs, for All of whom significantly lower kappa-scores In The complete cohort could Be found (*p* < 0.001 for intellectual disability, *p* = 0.013 for multifocal IEDs’ and *p* < 0.001 for abundant IEDs) (Figure 3). Multiple linear regression analysis Was used To test if intellectual disability, drug-resistant epilepsy, multifocal IEDs, and abundant IEDs significantly predicted lower kappa scores. The overall model Was statistically significant (F = 9.723, *p* < 0.001); however, only intellectual disability (*t* = −2.433, *p* = 0.016) and abundant IEDs (*t* = −4.167, *p* < 0.001) significantly predicted The lower kappa scores, But having DRE (*t* = 0.891, *p* = 0.891) or having multifocal IEDs (*t* = 0.403, *p* = 0.688) Did Not.

**Figure 4:**
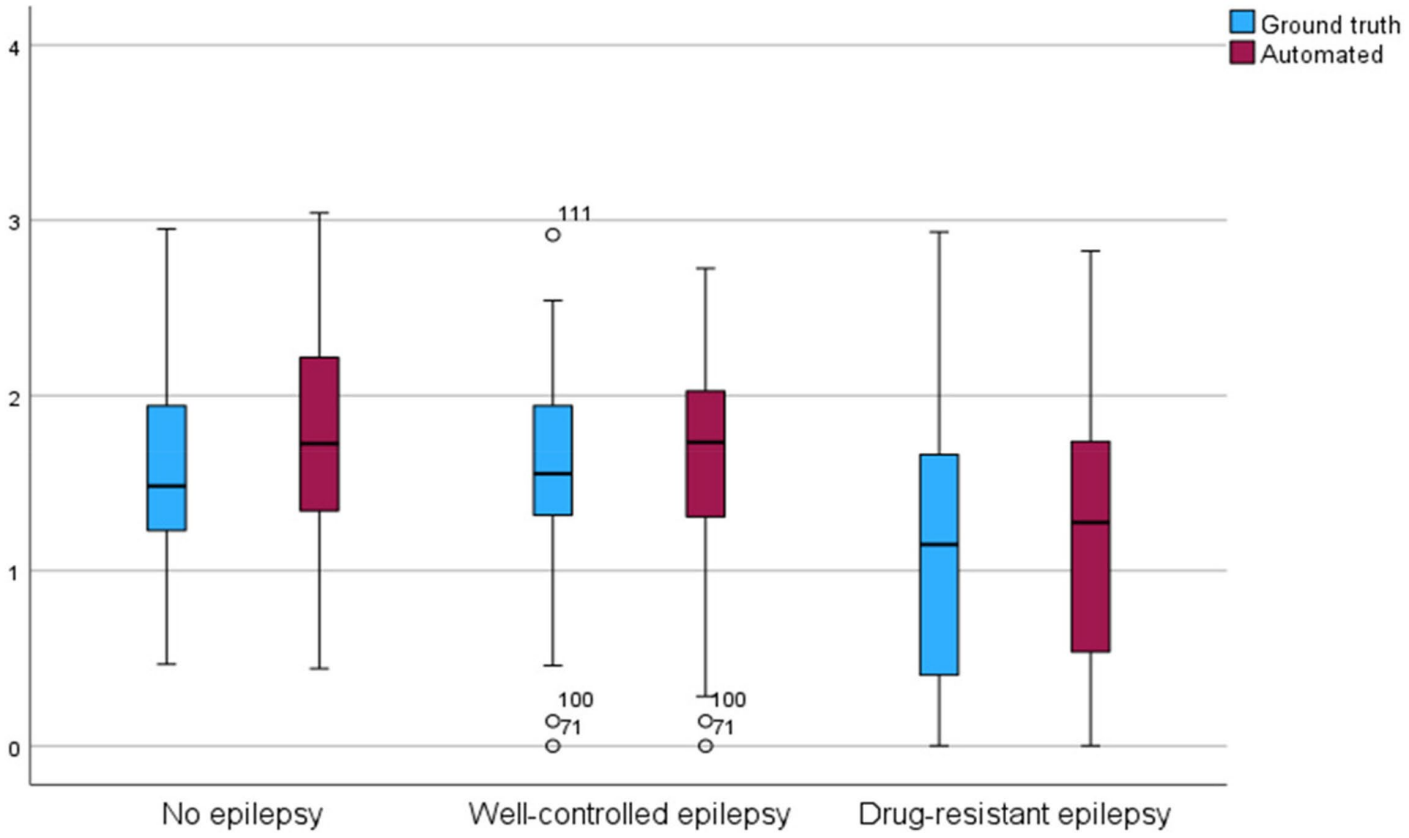
Box-plot comparing rapid eye movement (REM) sleep time of the ground truth and scored by the retrained algorithm. REM sleep time in hours on the y-axis.

In children with DRE, the amount of REM sleep time was significantly lower than in children with WCE and without epilepsy (P_DRE-control_ < 0.001, P_DRE-WCE_ < 0.001) (Figure 4). This finding could be reliably reproduced by the algorithm (P_DRE-control_ < 0.001, P_DRE-WCE_ < 0.001). The REM sleep time as calculated by the algorithm and with the manual hypnogram was significantly correlated with a Pearson correlation coefficient of *r* = 0.91 (*p* < 0.001).

## 4 Discussion

We investigated the performance of an automated sleep staging algorithm in a large pediatric cohort. The original SeqSleepNet, pre-trained on 200 healthy adults, already had good results in children without epilepsy and in children with WCE. After re-training the algorithm on the pediatric cohort, the agreement was substantial in all three of the groups, with Kappa scores of 0.79 in patients without epilepsy, 0.77 in patients with WCE, and 0.73 in patients with DRE. Nonetheless, in children with DRE, Kappa scores were significantly lower than in the other two groups. Furthermore, when looking at the different sleep stages separately, the scoring performance of the algorithm was good for all sleep stages except for stage N1.

When it comes to automated sleep staging based on EEG data in healthy adults, the problem can be considered solved (11), and as was shown by Phan et al. (12), the performance of automated sleep staging in healthy children was good. Furthermore, for neonatal sleep staging and sleep staging on stereo-EEG, there are promising results as well (19, 20).

There is limited data on automated sleep staging in children with epilepsy specifically. Skorucak et al. used a long short-term memory recurrent neural network to detect NREM sleep stages 2 and 3 in children with epilepsy and found a Kappa of 0.71 on a validation dataset (21). Since intellectual disability could have an independent effect on sleep architecture, research on patients with intellectual disability both with and without epilepsy is warranted. van den Broek et al. investigated an automated sleep scoring algorithm in patients older than 16 years old with intellectual disabiliy, including patients with epilepsy. The algorithm used ECG and respiratory effort as modalities (22). They found that the algorithm was least reliable in children who also had epilepsy, although these were also children with the most severe intellectual disabilities.

Our results showed significantly lower Kappa scores in the DRE group. Increased N1 time in this group could not fully explain this finding, but lower Kappa scores were found in children with intellectual disability and abundant IEDs. It must be said that scoring sleep stages on EEG signals is particularly difficult in children with abundant epileptiform activity, and one must be cautious in this population to assume that the ground truth is correct. One could argue that a more data-driven approach in automated sleep interpretation could be more informative in sleep and epilepsy research than classical sleep staging (23, 24). Sleep has a lot of temporal dynamics, which are not captured fully by the quite arbitrary classical sleep staging, divided into 30-s epochs. Frthermore, as it is clearly shown in the epilepsy population, pathology can disturb the sleep architecture in such a way that sleep stages, as such, might not be classifiable anymore. A microstructural approach [presence of spindles (25, 26), rapid eye movements (27) or cyclic alternating pattern (28)] or spectral power analysis are then helpful but are often still combined with sleep staging. Complete unsupervised learning could reveal intrinsic patterns, however, translating these patterns into clinically relevant information will be challenging. Furthermore, at a time when clinicians are only just getting to know artificial intelligence and their possibilities, it would still be valuable to have an easy way to quantify the sleep stages, something they have known and interpreted for the previous decades.

In some studies on sleep and epilepsy, the focus was mainly on N2-N3 sleep (21, 29). Although for N3 our algorithm showed a good performance, we feel that in the DRE group, encephalopathic slow waves and spike–wave discharges can be mistaken for N3 sleep, both by the human rater and algorithm at this point. A possible solution might be to train the algorithm to recognize the epileptiform activity from non-epileptiform slow waves or use a spike detector to remove all epileptiform activity in advance (21). If we assume that sleep, and most importantly non-REM sleep, is not erased but rather hijacked by epileptiform activity (30), important information might be lost. Studying slow wave characteristics and power spectral analysis throughout the night could be more insightful here, given the synaptic homeostasis hypothesis (21). Although we agree that slow-wave sleep is an important target for cognitive function, we feel a 5-stage sleep scoring including REM sleep is not to be neglected in children with epilepsy. There is sufficient evidence on the suppressive role of REM sleep on epileptiform activity (9, 27, 31–33). Our algorithm can reliably detect REM sleep and thus can be used to identify drug-resistant epilepsy patients with low REM sleep, which in the future could become a targeted treatment (34). Although not directly reported by the algorithm, all standard sleep parameters that rely on sleep staging, such as Total Sleep Time (TST, time spent in any sleep stage), Sleep Efficiency (SE, TST divided by time spent in bed), Sleep Onset Latency (SOL, time to first N1 sleep), and the percentage of TST in every sleep stage, can be calculated using a five-class sleep staging algorithm. Arousals and apneic events, however, are not detected.

In order to know how accurate the algorithm should be to be clinically useful, we can compare it to human inter-rater reliability. Lee et al. (7) performed a meta-analysis of 11 papers in which manual sleep staging was performed in an adult population without epilepsy. They reported a mean agreement of 0.83 between raters (Kappa 0.76), which our algorithm surpasses, at least for children with and without well-controlled epilepsy. The agreement of our algorithm in different sleep stages revealed the lowest value for N1, which is in line with inter-rater reliability between human raters. Furthermore, a high agreement for REM sleep has been described for human raters (6, 7). We assume that human inter-rater reliability in children with epilepsy, and even more so in drug-resistant epilepsy, is lower.

The algorithm can help accelerate sleep research in children with epilepsy. Since it uses only one EEG derivation to score sleep, it could potentially be used on reduced channel wearable EEG data, although we might need additional eye movements and chin EMG information for adequate results. Although commercially available non-EEG wearables can differentiate sleep and wake quite well, sleep staging performance is poor and not usable for clinical or research purposes (35). A wrist-worn wearable, detecting photoplethysmography and accelerometry was compared to polysomnography in an experimental setting, with a 4-class sleep staging kappa of 0.62 (36). This performance could be increased by combining behind-the-ear EEG with accelerometry.

However, there are some limitations to our study. Manual sleep scoring was performed by one experienced rater, hence no manual inter-rater reliability could be calculated for the manual sleep scoring. We acknowledge that the sleep parameters in some drug-resistant patients and the epileptic encephalopathies are so severely disturbed, that no human or algorithm could reliably say what sleep stage the patient is in. Before implementation, the algorithm should be validated in another scored dataset of children with DRE.

We conclude that the tested algorithm has a high sleep staging accuracy in children without epilepsy and with well-controlled epilepsy and can be used on hospital video-EEG data to estimate the hypnogram. Furthermore, when available in the future, it could be tested on a limited-channel wearable device. In our opinion, implementing such an algorithm in daily practice could detect children who suffer from sleep architecture changes more rapidly, and doing so could help in making treatment decisions. For drug-resistant patients with abundant nightly IEDs and intellectual disability, we feel the results should always be interpreted critically. Consequently, when sleep architecture disturbance is suspected from the clinical picture and the automated hypnogram used as a screening tool is indicative of this, a full polysomnography can be planned.

## Data availability statement

The raw data supporting the conclusions of this article will be made available by the authors, without undue reservation.

## Ethics statement

The studies involving humans were approved by Ethics Committee Research UZ/KU Leuven. The studies were conducted in accordance with the local legislation and institutional requirements. Written informed consent for participation in this study was provided by the participants’ legal guardians/next of kin.

## Author contributions

RP: Conceptualization, Data curation, Formal analysis, Investigation, Methodology, Visualization, Writing – original draft, Writing – review & editing. EH: Data curation, Formal analysis, Methodology, Software, Visualization, Writing – review & editing. LL: Funding acquisition, Supervision, Writing – review & editing. WP: Funding acquisition, Supervision, Writing – review & editing. MV: Funding acquisition, Supervision, Writing – review & editing. KJ: Conceptualization, Funding acquisition, Supervision, Writing – review & editing.
