# Supplementary material for: Automated sleep staging on reduced channels in children with epilepsy
Source: Front Neurol. 2024 May 10;15:1390465. doi: 10.3389/fneur.2024.1390465 (PMC11116721; doi:10.3389/fneur.2024.1390465)

Supplementary Figure 1: Confusion matrices showing the predictions by the network compared to the ground truth sleep stages. Left is the confusion matrix for the pre-trained algorithm, and right is the confusion matrix for the fine-tuned algorithm.


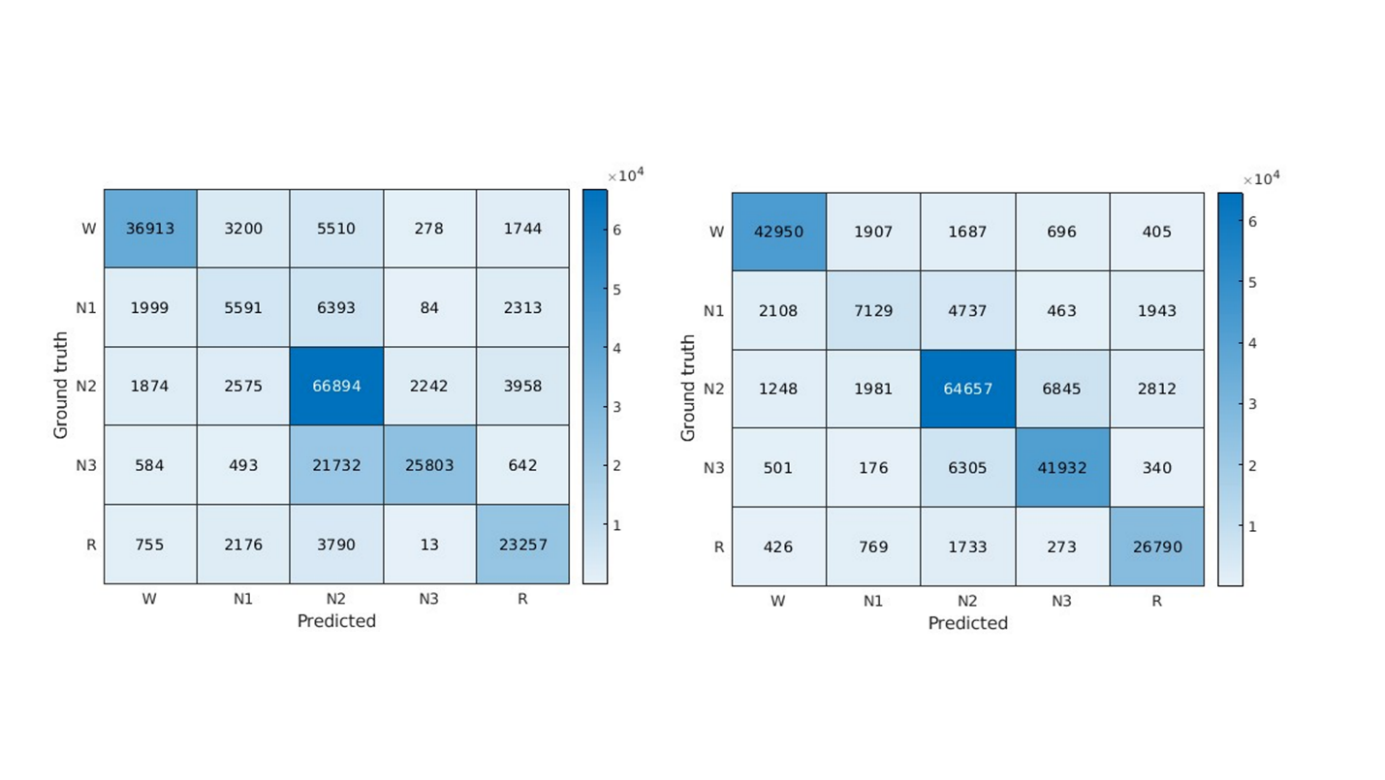

Supplement: Supplementary file 1 [file Data_Sheet_1.docx]
